# A curated, ontology-based, large-scale knowledge graph of artificial intelligence tasks and benchmarks

**DOI:** 10.1038/s41597-022-01435-x

**Published:** 2022-06-17

**Authors:** Kathrin Blagec, Adriano Barbosa-Silva, Simon Ott, Matthias Samwald

**Affiliations:** Medical University of Vienna, Center for Medical Statistics, Informatics and Intelligent Systems, Institute of Artificial Intelligence, Vienna, Austria

**Keywords:** Research data, Computational biology and bioinformatics

## Abstract

Research in artificial intelligence (AI) is addressing a growing number of tasks through a rapidly growing number of models and methodologies. This makes it difficult to keep track of where novel AI methods are successfully – or still unsuccessfully – applied, how progress is measured, how different advances might synergize with each other, and how future research should be prioritized. To help address these issues, we created the Intelligence Task Ontology and Knowledge Graph (ITO), a comprehensive, richly structured and manually curated resource on artificial intelligence tasks, benchmark results and performance metrics. The current version of ITO contains 685,560 edges, 1,100 classes representing AI processes and 1,995 properties representing performance metrics. The primary goal of ITO is to enable analyses of the global landscape of AI tasks and capabilities. ITO is based on technologies that allow for easy integration and enrichment with external data, automated inference and continuous, collaborative expert curation of underlying ontological models. We make the ITO dataset and a collection of Jupyter notebooks utilizing ITO openly available.

## Abstract (summary)

**Table:** 

Measurement(s)	Artificial Intelligence • Benchmark
Technology Type(s)	digital curation
Sample Characteristic - Location	Globally

## Background & Summary

The past decade led to substantial advances in Artificial intelligence (AI). Increases in computational capacity and the development of versatile machine learning models such as deep convolutional neural networks or the transformer architecture made it possible to tackle a wide variety of tasks that were previously deemed intractable^1,2^.

According to the 2021 AI Index Report published by the AI Index Steering Committee of the Human-Centered AI Institute (Stanford University), the number of AI conference publications increased fourfold between the years 2000 and 2019, while the number of AI journal publications grew from roughly 10,000 in the year 2000 to more than 120,000 in the year 2019^3^. Similarly, AI-related publications on the pre-print server arXiv increased more than sixfold within only five years from 2015 to 2020^3^.

This ever-growing amount of research on AI methods, models, datasets and benchmarks makes it difficult to keep track of where novel AI methods are successfully (or still unsuccessfully) applied, how quickly progress happens, and how different AI capabilities interrelate and synergize. This complexity is amplified by the great variety of AI data modalities (e.g., natural language, images, audio, structured data) and application domains (e.g., web search, biology, medicine, robotics, security, advertising). Furthermore, real-world AI systems and the tasks they address are tightly embedded in complex systems of data creation/consumption, non-AI algorithms and social processes. Understanding AI and its global impact requires the creation of rich models that integrate data from these adjacent knowledge domains.

To help address these issues, we introduce the Intelligence Task Ontology and Knowledge Graph (ITO), a comprehensive, richly structured and manually curated data resource on artificial intelligence tasks, benchmark results and performance metrics. ITO is realized as an ontology-backed knowledge graph^4^ based on standards minted by the World Wide Web Consortium (W3C). Data are represented through the Resource Description Framework (RDF)^5^ and Web Ontology Language (OWL)^6^ standards and can be queried through the SPARQL graph query language^7^. These standards have a long history of application in other domains requiring complex knowledge representation and integration, such as biomedical research^8–10^.

The following desiderata guided the creation of ITO:

- Manual curation of AI task classification hierarchies and performance metrics, enabling more precise analyses.
- Representing data as a graph, facilitating network-based query and analysis.
- Allowing for easy integration and enrichment with external data, as well as simple extensibility for modeling related knowledge domains from other domains.
- Allowing for automated deductive inference and automated knowledge base consistency checking.
- Allowing for ongoing, collaborative expert curation of underlying ontological models.

ITO allows for capturing rich relationships between AI processes, models, datasets, input and output data types (e.g., text, video, audio) metrics, performance results and bioinformatics processes. This enables a more in-depth tracking of progress over time, such as analysing how progress trajectories on various classes of tasks compare to each other across different dimensions.

In its current version (v1.01), ITO encompasses more than 50,000 data entities and 9,000 classes.

### Exemplary use cases

The primary aim of ITO is to enable **‘meta-research’** concerned with studying scientific research itself in terms of its methods, reporting, evaluation and other aspects to increase the quality of scientific research^11^. The value of ITO for meta-research can be exemplified based on two recent studies utilizing the resource.

As a first step in creating insights from ITO, our group analysed the prevalence of performance metrics currently used to measure progress in AI using data on more than 30,000 performance results across more than 2,000 distinct benchmark datasets. To increase data quality and enable a thorough analysis, we conducted extensive manual curation and annotation of the raw data as part of integrating it into the ontology^12^.

In another recent study, we explored and mapped AI capability gains over time across 16 main research areas (e.g., computer vision, natural language processing, graph processing), further breaking down capability gain by sub-processes described in the curated AI process hierarchy (Barbosa-Silva *et al*., manuscript in preparation). This analysis made use of both the curated AI process class structure, as well as the cleaned and normalized performance metrics data.

Besides providing a knowledge graph for meta-research, the ontology of ITO can be **utilized as a taxonomic resource for annotating and organizing** information in the AI domain. For example, in recent work, our group conducted a systematic review of literature and online resources to create a catalogue of AI datasets and benchmarks for medical decision making^13,14^. Identified datasets and benchmarks were manually annotated for meta-information, such as targeted tasks, data types and evaluation metrics. This manual curation process was greatly simplified by using the taxonomic structures provided by ITO together with the OntoMaton ontology annotation widget^15^.

Finally, the ITO knowledge network can serve as a practice-focused resource that allows developers to **find, compare and select AI models to address complex use-cases** for certain defined tasks, data types and application domains.

## Methods

Benchmark result and initial task description data was drawn from the ‘Papers with code’ (PWC, https://paperswithcode.com) repository. PWC is the largest repository of AI benchmark data currently available. It is a web-based open platform that contains information on more than 5,000 benchmarks and 50,000 publications. PWC data was collected by combining automatic extraction from arXiv submissions and manual crowd-sourced annotation of benchmark results.

To create the initial version of ITO, PWC data was imported and converted to RDF/OWL with a Python script. After initial data import from PWC, ITO underwent extensive further manual curation. Collaborative manual curation was done using WebProtégé^16^. The top-level class hierarchy of AI processes was derived from the top-level classification of benchmarks on the Papers With Code platform. As an established community of practice, we considered this source to be a good starting point. We were unable to identify other existing resources with a more well-grounded top-level classification of AI tasks. Curation was conducted by two experts in the AI/machine learning domain (KB, MS) over the course of several months. In this process, tasks and benchmarks were systematised and mapped to AI processes. Where appropriate, classes and properties from the following established ontologies were re-used: the EMBRACE Data And Methods (EDAM) ontology^17^, the Open Biomedical Ontologies (OBO) in OWL ontology^18^, the Dublin Core schema^19^ and the Friend of a Friend (FOAF) ontology^20^.

The scripts and overall workflow created allow for repeated updating and incremental curation of data over time, so that ITO can be kept up-to-date as benchmark result data in Papers With Code (and potentially other data repositories) keeps evolving. Novel classes and properties identified and imported through our automated scripts were put under the “Meta: Class requiring curation” class or the “Meta: Data property requiring curation” property to flag them for further manual curation by our curators. This way, novel data could be reflected by ITO quickly, while allowing for ongoing manual curation and improvement of the model.

### Process-centric modeling of AI tasks, benchmarks and data

Table 1 provides an overview of the main classes and properties used to capture knowledge in ITO.

**Table 1:** Overview of the main classes and properties.

Main classes	
AI process	Subclasses of *AI process* represent a wide variety of AI processes and tasks, such as natural language processing, image classification or link prediction.
Data	Subclasses of the *Data* are primarily used to represent different kinds of data that AI processes deal with, such as text, graphs or visual data.
- Benchmark dataset	*Benchmark dataset* is an indirect subclass of *Data*. Instances of the *Benchmark dataset* class are used to represent individual benchmark datasets.
- Article	*Article* is an indirect subclass of *Data*. Instances of the *Article* class are used to represent research articles associated with benchmark results.
edam:Data format	Subclasses of *Data format* are used to represent different data that different kinds of data can be represented in, such as XML or PNG.
Software	Instances of *Software* represent AI models used in benchmark experiments.
Topic	Subclasses of the *Topic* class are used to further describe the topics that certain datasets or AI processes deal with, such as different knowledge domains, scientific disciplines or languages.
**Main properties**	
involves data	The *involves data* object property relates AI processes to the data that are used or generated by the process.
- edam:has input	The *has input* object property is a subproperty of *involves data*, it relates AI processes to the data that are used as input for the process.
- edam:has output	The *has input* object property is a subproperty of *involves data*, it relates AI processes to the data that are the output of the process.
Performance measure	The rich hierarchy of subproperties of the *Performance measure* datatype property represent quantitative measures of AI model performance on benchmarks, such as accuracy, F1 score or Recall-Oriented Understudy for Gisting Evaluation (ROUGE) score.
edam:has topic	The *has topic* object property relates *Topic* to *AI process* or *Data* subclasses.
edam:is format of	The *is format of* object property *Data format* to *Data* subclasses.
rdfs:seeAlso	The *seeAlso* annotation property is used to relate benchmark results to the research articles in which results were reported, the model utilized for generating the benchmark result and other, secondary information.
foaf:page	The *page* annotation property is primarily used to link research articles to the URLs where they can be retrieved from.
obo:date	The *date* annotation property is used to provide the dates of publication of specific benchmark results or research articles.
obo:creation date	The *creation date* annotation property is used to represent the time of the creation of ontology/knowledge graph resources (e.g., when an entity was generated through the automated import procedure).

Prefixes denote classes or entities derived from established vocabularies, i.e., EDAM ontology (edam), Resource Description Format Schema (rdfs), Open Biomedical Ontologies (obo) and Friend of a Friend (foaf).

In ITO, an ‘AI process’ is defined as a process that can be carried out (or can partially be carried out) by an AI system. We chose to focus the ontological representation on processes rather than potential alternative notions like ‘tasks’, since existing foundational and domain ontologies provide clear definitions for processes, while possible alternatives are less well-represented or researched. Individual benchmark results are represented as instances of the subclasses of ‘AI process’, i.e., they are conceptualized as concrete AI processes that resulted in certain outcomes and measurements. This conceptualization also allows for potential future extensions of the ontology and knowledge graph to interlink AI processes with other processes, such as the processes that constitute scientific research, bioinformatics operations or human cognition.

AI processes are organised into 16 major parent classes, e.g, ‘Natural Language Processing’, ‘Vision process’ or ‘Audio process’, and further into a hierarchy of subclasses informed by the common terminology of the respective research field. For example, the branch of the class ‘Natural language processing’ was informed both by terminology and taxonomies used in the fields of linguistics and machine learning.

In the current version of ITO, AI processes are represented through a simple, asserted polyhierarchy. Since some AI tasks are cross-modal, child classes can have more than one parent class (multiple inheritance). For example, the process ‘Image question answering’, which is concerned with answering questions based on the semantic content of an image, has both ‘Natural language processing’ and ‘Vision process’ as its superclasses. This application-centric modelling approach was deemed appropriate for currently targeted use cases of ITO, but we plan to utilize more elaborate ontological modeling in future work (e.g., breaking up the process class hierarchy into several complementary axes and making greater use of logical class definitions).

Instances of AI models, e.g. ‘BERT’ or convolutional neural networks, and benchmark datasets, e.g., ‘ImageNet’, are modelled via the ‘Data’ and ‘Software’ branches of ITO.

Individual benchmark results were captured as instances of the respective benchmark class and connected to the respective dataset via a ‘has_input’ annotation. Performance measures (e.g., F1 score) were modelled through a hierarchy of data properties.

Similar to the AI process classes, performance measure properties underwent extensive manual curation. The original data obtained from PWC contained more than 800 different strings representing metric names that were used by human annotators on the PWC platform to add model performance results. Based on this raw list containing more than 60 naming variations of the same metric in some cases, we created a canonical hierarchy of performance measures, and mapped the strings accordingly. To select the canonical names for the metrics, a preference was given to Wikipedia article titles whenever sensible. Additional details and complexities of this process are described in previous work^12^.

**Fig. 1:**
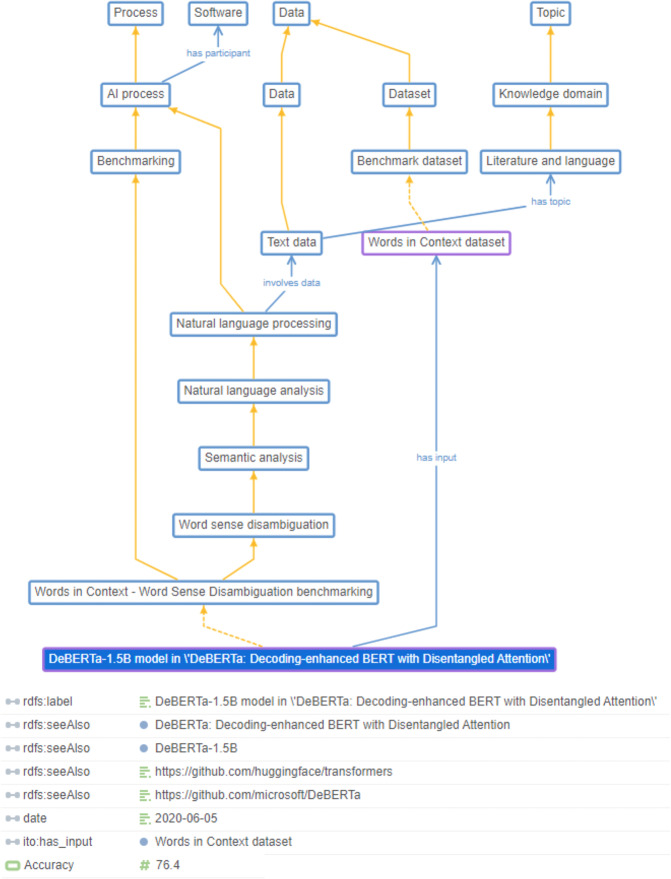
Example of a benchmark result for a specific model (‘DeBERTa-1.5B’) on a specific dataset (‘Words in Context’, Word sense disambiguation) embedded in ITO. Solid orange lines represent subclass relations, dashed orange lines represent instance relations.

Figure 1 shows an example of a benchmark result achieved by a specific *model* on a specific *dataset* for a specific *AI process* embedded in ITO.

## Data Records

The ITO dataset is made available as a single OWL (Web Ontology Language) file. The ITO model is available as an OWL (Web Ontology Language) file. The latest version of ITO is available on Zenodo (10.5281/zenodo.5561989)^21^, GitHub (https://github.com/OpenBioLink/ITO) and BioPortal (https://bioportal.bioontology.org/ontologies/ITO). The ontology file is distributed under a CC-BY-SA license.

The current version of ITO (v1.01) encompasses more than 50,000 individuals across more than 9,000 classes. Additional basic metrics are shown in Table 2.

**Table 2:** Basic ontology metrics of ITO (v1.01).

Entities	Count
Total triples (i.e. edges in the RDF graph)	685,560
Logical axioms count	116 828
Classes (total)	9,037
Classes (AI process classes)	1,100
Individuals	50,826
Object properties	16
Data properties (i.e. AI performance measures)	1,995
Annotation properties	32
Maximum depth	11
DL expressivity	ALCHOI(D)

**Fig. 2:**
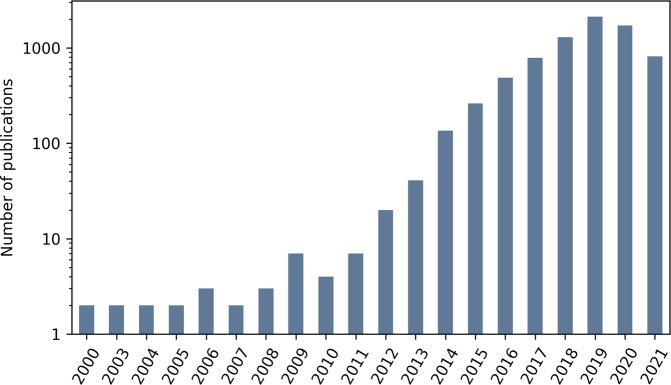
Number of papers covered by ITO per year. The y-axis is scaled logarithmically. Publications of the year 2021 are covered until the latest import in August 2021.

In total, ITO captures more than 26,000 benchmark results across more than 3,633 benchmark datasets covering the years 2000 to 2021 (see Table 3 and Fig. 2).

**Table 3:** Content metrics (v1.1).

	Count
Total number of papers covered	7,649
Time span of publications covered	2000–8/2021
Total number of benchmark results	26,495
Total number of benchmark datasets	3,633

**Fig. 3:**
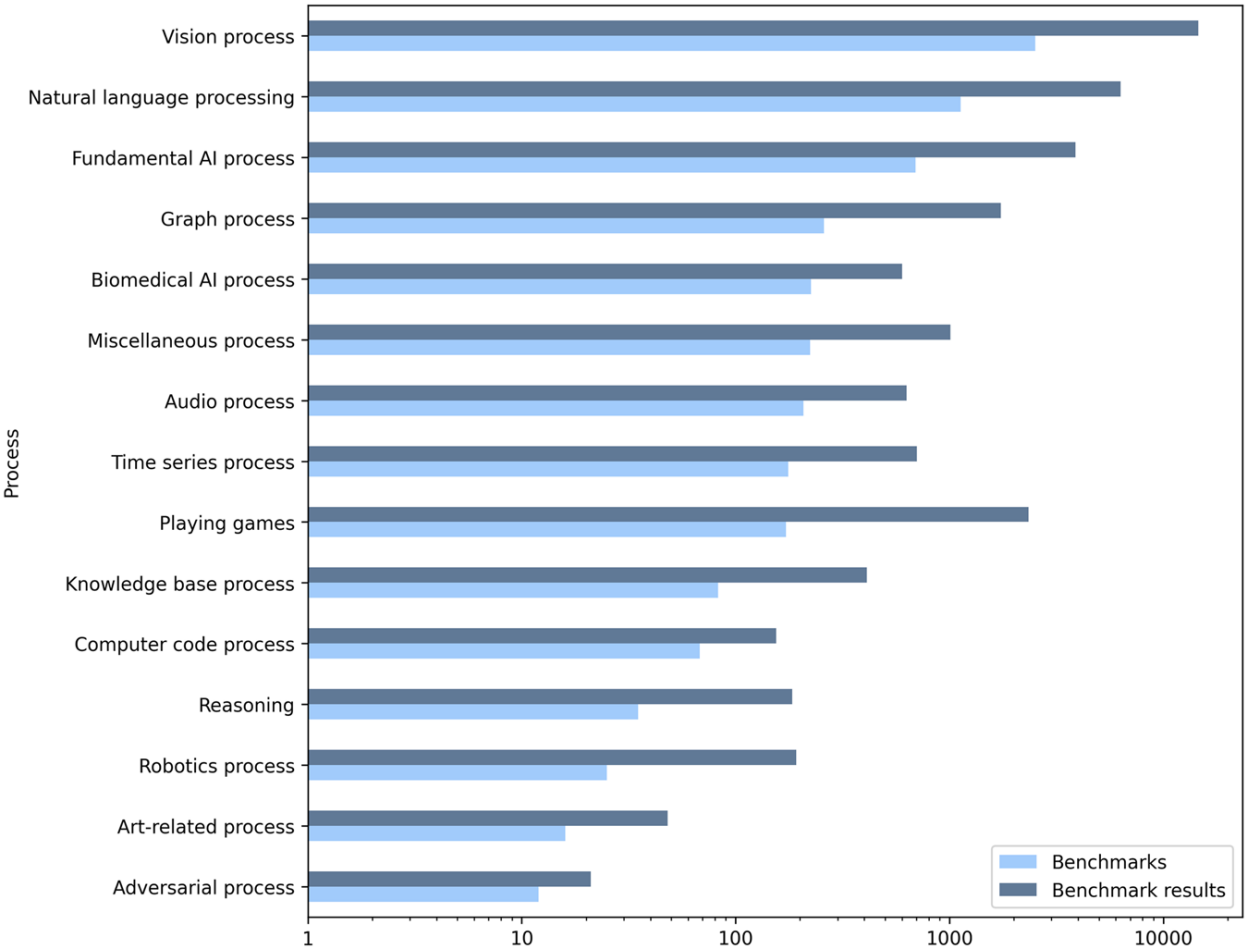
Number of distinct benchmarks and benchmark results per ‘AI process’ class. The x-axis is scaled logarithmically.

**Fig. 4:**
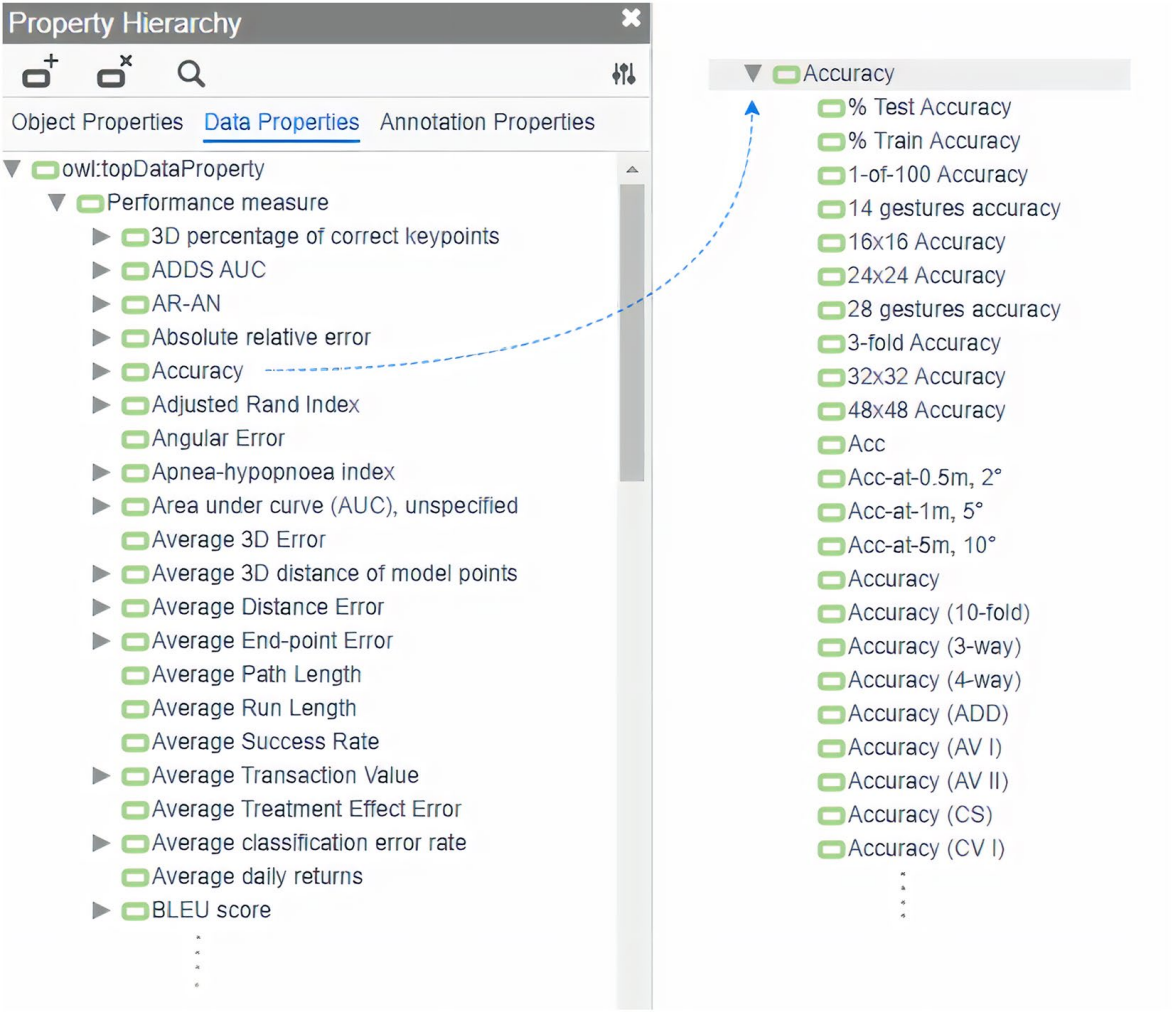
Performance measures property hierarchy. The left side of the image shows an excerpt of the list of performance metric properties; the right side shows an excerpt of the list of subclasses for the parent class ‘accuracy’.

Figure 3 shows the 16 parent process classes, e.g, ‘Natural Language Processing’, ‘Computer vision’ or ‘Audio process’ that are used to map AI processes in ITO together with the number of distinct benchmarks and benchmark results per process. An excerpt of the curated performance measure hierarchy is displayed in Fig. 4.

## Technical Validation

Validation and evaluation of a knowledge graph and/or ontology aims to assess whether the resource adequately and accurately covers the domain it intends to model, and whether it enables an efficient execution of the tasks it was designed for.

Commonly used criteria to evaluate ontologies based on these aspects include *accuracy*, *clarity*, *completeness*, *conciseness*, *adaptability*, *computational efficiency* and *consistency*^22^.

*Accuracy* indicates whether the definitions and descriptions of elements in an ontology are correct. *Clarity* measures whether the ontology’s elements are clearly defined and labeled, and understandable for the user. Achieving high accuracy and clarity was ensured in ITO through an extensive manual curation period lasting several months.

The criterion of *completeness* is concerned with whether the domain to be modeled is adequately covered by the ontology, while *conciseness* indicates to which extent the ontology covers only elements relevant to the domain. Both criteria are ensured in ITO through the bottom-up development approach that makes use of existing data (i.e., benchmarks extracted from preprint servers) and concepts relevant to the domain of AI processes instead of a top-down approach that starts with a blank slate. Relying on existing data sources, such as the PWC database that combines automated extraction of benchmarks from papers on preprint servers and crowd-sourced annotation by several thousands of contributors enables high domain coverage. Completeness was further tested by using ITO to annotate a collection of over 450 datasets and AI benchmarks in the biomedical domain, with ITO being found to cover all required concepts to annotate all datasets^13^.

*Adaptability* is concerned with whether the ontology meets the requirements defined by the range of use cases for which it was built. The practical usability of ITO for its intended applications has been validated within two recently conducted studies (Barbosa-Silva *et al*., manuscript in preparation)^12^.

*Computational efficiency* indicates whether the ontology’s anticipated tasks can be fulfilled within reasonable time and performance frames using the available tools. Even complex queries related to the use cases described above can be executed within a few seconds on standard hardware when using the high-performance Blazegraph graph database.

Finally, *consistency* requires the ontology to be free from any contradictions. Internal consistency was checked using Protégé v5.5.0 and the elk 0.4.3 reasoner^23,24^.

Furthermore, common pitfalls in ontology design and creation have been described, which, for example, include the creation of unconnected ontology elements, missing human readable annotations or cycles in class hierarchies^25–28^. ITO was checked for these with the ontology quality checking tool ‘OOPS!’^27^, and identified issues were resolved.

Ontology evaluation metrics were calculated with the Ontometrics tool^29^ and were used for ontology quality evaluation following the example of Carriero *et al*.^30^. Ontology metrics are reported in Table 4.

**Table 4:** Ontology evaluation metrics.

*Schema metrics*	
**Attribute richness** – Average number of attributes per class; the more attributes, the more knowledge the ontology conveys	0.22
**Inheritance number** – Average number of subclasses per class; an indication of how well knowledge is grouped	1.73
**Relationship richness** – Number of non-inheritance relationships (e.g. not subClassOf) divided by total number of relationships	0.002
**Axiom/class ratio** – Ratio between axioms and classes	75.62
***Knowledge base metrics***	
**Average population** – Number of instances of the knowledge base divided by number of classes defined in the ontology schema	5.62
**Class richness** – Number of non-empty classes (classes with instances) divided by the total number of classes.	0.49
***Graph/class hierarchy metrics***	
**Number of leaf classes (NoL)** – classes with no subclasses	7328
**Average depth**	5.36
**Maximal depth**	11
**Absolute breadth**	18 849
**Average breadth**	7.37
**Maximal breadth**	4590
**Absolute sibling cardinality** – the number of sibling classes in the hierarchy	9037
**Ratio of leaf fanoutness** – Number of leaf classes divided by total number of classes	0.81
**Tangledness** – Degree of multihierarchical nodes (i.e., nodes with multiple super classes) in the class hierarchy	0.68

The *inheritance number* of 1.73 is low, suggesting that ITO is a deep ontology, i.e. the class hierarchy is well grouped and covers the domain in a detailed manner. The *relationship richness* as calculated by the Ontometrics algorithm of 0.002 is low, which, however is due to the fact that the vast majority of relationships in ITO are captured at the level of OWL individuals rather than classes. The axiom/class ratio is high, indicating a richly axiomatized ontology. The *average population* number of 5.62 indicates a good balance between the count of individuals (i.e., mostly benchmark results) and the number of classes in the class hierarchy used to structure those results. The *class richness* of 0.49 suggests that roughly half of the classes in the ontology are not instantiated by individuals; this is due to *Data*, *Data format* and *Topic* branches of the ontology that are primarily used for defining attributes of other classes, rather than being instantiated themselves. The average depth depth value of 5.36 is within the normal ranges for an ontology of the given size. The maximal breadth and absolute sibling cardinality of 4590 and 9037 are very high. This is caused by the modeling decision of creating a process class called *Benchmarking*, which is the direct superclass of the large number of classes representing benchmarks in the ontology. This design choice also led to a high tangledness metric, i.e. a large number of classes with multiple superclasses, since benchmark classes have both a specific AI task and the *Benchmarking* class as direct superclasses. While this particular design choice deviates from best practices of ontology design, it proved favorable for ease of querying the ontology, which was an important design goal.

### Other data sources and related work

Besides PWC, we also investigated some other projects aiming to track global AI tasks, benchmarks and state-of-the-art results have been initiated in recent years as potential data sources. Among these, the *Aicollaboratory*^31^ and *State of the art AI* (https://www.stateoftheart.ai/) stood out as the most comprehensive and advanced resources.

‘AIcollaboratory’ is a data-driven framework enabling the exploration of progress in AI. It is based on data from annotated AI papers and open data from, e.g., PWC, AI metrics and OpenML. Similar to the projects described above, benchmark results are organized hierarchically and can be compared per task. In addition, the platform provides summary diagrams that combine all benchmark results per top-level task class, e.g., ‘Natural language processing’ and display progress over time. We found that relevant data in AIcollaboratory were already covered by PWC, and that the project did not seem to be actively maintained at the moment.

‘State of the art AI’ collects AI tasks and datasets, models and papers building on data from PWC, arXiv, DistillPub and others. Similar to PWC, it organises AI tasks, allows for a comparison of results per task, and makes them available on a web-based platform. However, data are not available for download at the time of this writing, and relevant data were already covered by PWC.

There are some ontologies and taxonomies that are related to ITO. The *Computer Science Ontology* (CSO)^32^ is a large-scale ontology created through literature mining that captures research areas and their relations in computer science. *WikiCSSH* provides a large-scale, hierarchically organized vocabulary of subjects in computer science that was derived from Wikipedia^33^. Compared to ITO, CSO and WikiCSSH have lower coverage of the domain of AI tasks. Outside of the domain of computer science, the *Cognitive Atlas Ontology* provides concepts of human cognition that partially overlap with concepts from AI^34^.

There are several related projects that aim to capture scientific results through knowledge graphs. The *Artificial Intelligence Knowledge Graph* (AI-KG) contains a large collection of research statements mined from AI manuscripts^35^. The *Open Research Knowledge Graph* (ORKG)^36^ captures research statements across multiple scientific domains. The *Academia/Industry DynAmics* (AIDA) Knowledge Graph describes 21 million publications and 8 million patents and utilizes CSO for annotations.

There are also multiple partially related initiatives towards creating large, integrated knowledge graphs in the life sciences. The decentralized *nanopublications* infrastructure that captures and integrates research statements and their provenance, particularly in the domain of life sciences^37^. More centralized ontology-based knowledge graphs that were recently published include *OpenBioLink*^38^, *Hetionet*^39^ and *PheKnowLator*^40^.

### Maintenance and future development

To ensure content validity and keeping up with the fast-paced developments in the field of AI, newly available data will be periodically imported. Furthermore, the underlying ontological model will be subject to continuous refinement, and future developments will also focus on creating mappings between ITO and other thematically relevant ontologies and knowledge graphs, particularly AI-KG, ORKG and CSO.

## Usage Notes

A wide variety of frameworks for OWL, RDF and the SPARQL graph query language can be used to access and query the ontology. Our recommendations for efficient processing include the graph database Blazegraph (https://blazegraph.com) for both simple and complex queries requiring high performance, and the Owlready2 Python library (https://pypi.org/project/Owlready2/) for simple queries and OWL reasoning.

Example Jupyter notebooks for querying the ontology using the libraries mentioned above can be found in the associated Github repository (https://github.com/OpenBioLink/ITO) in the folder ‘notebooks’ (e.g., ‘descriptive_statistics_v1.0’ and ‘trajectories_notebooks’).

To view and edit the ontology, the Protégé ontology editor (https://protege.stanford.edu/) can be used^23^. Furthermore, the class structure of ITO can be browsed online via BioPortal (https://bioportal.bioontology.org/ontologies/ITO).

## Data Availability

Code to generate the summary statistics are available from the Github repository in the folder ‘notebooks’: https://github.com/OpenBioLink/ITO. The entire Github repository for the v1.01 release is archived on Zenodo^41^: 10.5281/zenodo.6566103.

## References

[1] Krizhevsky, A., Sutskever, I. & Hinton, G. E. ImageNet classification with deep convolutional neural networks. (2012).

[2] Vaswani, A. *et al*. Attention Is All You Need. *arXiv* (2017).

[3] Zhang, D. *et al*. *The AI Index 2021 Annual Report*. https://aiindex.stanford.edu/wp-content/uploads/2021/03/2021-AI-Index-Report_Master.pdf (2021).

[4] Hogan, A. *et al*. Knowledge Graphs. *arxiv* (2020).

[5] RDF 1.1 Concepts and Abstract Syntax. https://www.w3.org/TR/rdf11-concepts/.

[6] OWL 2 Web Ontology Language Primer (Second Edition). https://www.w3.org/TR/owl2-primer/.

[7] SPARQL 1.1 Overview. https://www.w3.org/TR/sparql11-overview/.

[8] Ruttenberg, A. *et al*. Advancing translational research with the Semantic Web. *BMC Bioinformatics* **8** Suppl 3, S2 (2007). 10.1186/1471-2105-8-S3-S2 PMC1892099 17493285

[9] Dumontier M (2014). The Semanticscience Integrated Ontology (SIO) for biomedical research and knowledge discovery. J. Biomed. Semantics.

[10] Auer, S. *et al*. Towards a knowledge graph for science. in *Proceedings of the 8th International Conference on Web Intelligence, Mining and Semantics - WIMS ’18* (eds. Akerkar, R. *et al*.) 1–6, 10.1145/3227609.3227689 (ACM Press, 2018).

[11] Ioannidis JPA (2018). Meta-research: Why research on research matters. PLoS Biol..

[12] Blagec, K., Dorffner, G., Moradi, M. & Samwald, M. A critical analysis of metrics used for measuring progress in artificial intelligence. https://arxiv.org/abs/2008.02577 (2020).

[13] Blagec, K., Kraiger, J., Frühwirt, W. & Samwald, M. Benchmark datasets driving artificial intelligence development fail to capture the needs of medical professionals. *arXiv* (2022). 10.1016/j.jbi.2022.104274 36539106

[14] Blagec K, Kraiger J, Samwald M (2021). Zenodo.

[15] Maguire E, González-Beltrán A, Whetzel PL, Sansone S-A, Rocca-Serra P (2013). OntoMaton: a bioportal powered ontology widget for Google Spreadsheets. Bioinformatics.

[16] Horridge, M., Gonçalves, R. S., Nyulas, C. I., Tudorache, T. & Musen, M. A. WebProtégé: A Cloud-Based Ontology Editor. in *Companion Proceedings of The 2019 World Wide Web Conference on - WWW ’19* (eds. Liu, L. & White, R.) 686–689, 10.1145/3308560.3317707 (ACM Press, 2019).

[17] Ison J (2013). EDAM: an ontology of bioinformatics operations, types of data and identifiers, topics and formats. Bioinformatics.

[18] Tirmizi, S. H. *et al*. Mapping between the OBO and OWL ontology languages. *J. Biomed. Semantics* **2** Suppl 1, S3 (2011). 10.1186/2041-1480-2-S1-S3 PMC3105495 21388572

[19] Dublin Core Metadata Initiative. Dublin Core Metadata Element Set, Version 1.1. (2012).

[20] Graves M, Constabaris A, Brickley D (2007). FOAF: connecting people on the semantic web. Cataloging & Classification Quarterly.

[21] Samwald M, Blagec K (2021). Zenodo.

[22] Raad, J. & Cruz, C. A survey on ontology evaluation methods. in *Proceedings of the 7th International Joint Conference on Knowledge Discovery, Knowledge Engineering and Knowledge Management* 179–186, 10.5220/0005591001790186 (SCITEPRESS - Science and and Technology Publications, 2015).

[23] Musen MA, Protégé Team. (2015). The Protégé Project: A Look Back and a Look Forward. AI Matters.

[24] Kazakov Y, Krötzsch M, Simančík F (2014). The Incredible ELK. J. Autom. Reasoning.

[25] Aguado-de-Cea G, Montiel-Ponsoda E, Poveda-Villalón M, Giraldo-Pasmin OX (2015). Lexicalizing ontologies: the issues behind the labels. Procedia - Social and Behavioral Sciences.

[26] Gómez-Pérez, A. Evaluation of Taxonomic Knowledge in Ontologies and Knowledge Bases. in *Proceedings of the 12th Banff Knowledge Acquisition for Knowledge-Based Systems Workshop*, *Banff, Alberta, Canada* (1999).

[27] Poveda-Villalón M, Gómez-Pérez A, Suárez-Figueroa MC (2014). OOPS! (ontology pitfall scanner!). Int. J. Semant. Web Inf. Syst..

[28] Vrandečić, D. Ontology Evaluation. in *Handbook on Ontologies* (eds. Staab, S. & Studer, R.) 293–313, 10.1007/978-3-540-92673-3_13 (Springer Berlin Heidelberg, 2009).

[29] Lantow, B. OntoMetrics: Putting Metrics into Use for Ontology Evaluation. in *Proceedings of the 8th International Joint Conference on Knowledge Discovery, Knowledge Engineering and Knowledge Management* 186–191, 10.5220/0006084601860191 (SCITEPRESS - Science and and Technology Publications, 2016).

[30] Carriero VA (2021). Pattern-based design applied to cultural heritage knowledge graphs. SW.

[31] Martínez-Plumed, F., Hernández-Orallo, J. & Gómez, E. Tracking AI: The Capability Is (Not) Near. in *Proceedings of ECAI 2020* 2915–2916 (IOS Press, 2020).

[32] Salatino AA (2020). The Computer Science Ontology: A Comprehensive Automatically-Generated Taxonomy of Research Areas. Data Intelligence.

[33] Han, K., Yang, P., Mishra, S. & Diesner, J. WikiCSSH: Extracting Computer Science Subject Headings from Wikipedia. in *ADBIS, TPDL and EDA 2020 common workshops and doctoral consortium: international workshops: DOING, MADEISD, SKG, BBIGAP, SIMPDA, aiminscience 2020 and doctoral consortium, lyon, france, august 25–27, 2020, proceedings* (eds. Bellatreche, L. *et al*.) **vol. 1260** 207–218 (Springer International Publishing, 2020).

[34] Poldrack RA (2011). The cognitive atlas: toward a knowledge foundation for cognitive neuroscience. Front. Neuroinformatics.

[35] Dessì, D. *et al*. AI-KG: An Automatically Generated Knowledge Graph of Artificial Intelligence. in *The semantic web – ISWC 2020*: *19th international semantic web conference*, *athens, greece, november 2–6, 2020, proceedings*, *part II* (eds. Pan, J. Z. et al.) **vol. 12507** 127–143 (Springer International Publishing, 2020).

[36] Jaradeh, M. Y. *et al*. Open research knowledge graph: next generation infrastructure for semantic scholarly knowledge. in *Proceedings of the 10th International Conference on Knowledge Capture - K-CAP ’19* 243–246, 10.1145/3360901.3364435 (ACM Press, 2019).

[37] Kuhn, T. *et al*. Nanopublications: A Growing Resource of Provenance-Centric Scientific Linked Data. in *2018 IEEE 14th International Conference on e-Science (e-Science)* 83–92, 10.1109/eScience.2018.00024 (IEEE, 2018).

[38] Breit A, Ott S, Agibetov A, Samwald M (2020). OpenBioLink: a benchmarking framework for large-scale biomedical link prediction. Bioinformatics.

[39] Himmelstein, D. S. *et al*. Systematic integration of biomedical knowledge prioritizes drugs for repurposing. *eLife* **6** (2017). 10.7554/eLife.26726 PMC5640425 28936969

[40] Callahan TJ, Tripodi IJ, Hunter LE, Baumgartner WA (2020). A Framework for Automated Construction of Heterogeneous Large-Scale Biomedical Knowledge Graphs. BioRxiv.

[41] Samwald M (2022). Zenodo.

